# Heterotypic breast cancer model based on a silk fibroin scaffold to study the tumor microenvironment

**DOI:** 10.18632/oncotarget.23574

**Published:** 2017-12-22

**Authors:** Ewelina Dondajewska, Wojciech Juzwa, Andrzej Mackiewicz, Hanna Dams-Kozlowska

**Affiliations:** ^1^ Chair of Medical Biotechnology, Poznan University of Medical Sciences, Poznan 60-806, Poland; ^2^ Department of Biotechnology and Food Microbiology, Poznan University of Life Sciences, 60-627 Poznan, Poland; ^3^ Department of Diagnostics and Cancer Immunology, Greater Poland Cancer Centre, Poznan 61-866, Poland

**Keywords:** breast cancer, 3D tumor model, tumor microenvironment, cells co-culture, silk fibroin scaffold

## Abstract

An intensive investigation of the development of *in vitro* models to study tumor biology has led to the generation of various three-dimensional (3D) culture methods that better mimic *in vivo* conditions. The tumor microenvironment (TME) is shaped by direct interactions among cancer cells, cancer-associated cells and the extracellular matrix (ECM). Recognizing the need to incorporate both tissue dimensionality and the heterogeneity of cells, we have developed a 3D breast cancer model. NIH3T3 fibroblasts and EMT6 breast cancer cell lines were seeded in various ratios onto a silk fibroin scaffold. The porosity of the silk scaffold was optimized to facilitate the growth of cancer cells. EMT6 and NIH3T3 cells were modified to express GFP and turboFP635, respectively, which enabled the direct analysis of the cell morphology and colonization of the scaffold and for the separation of the cells after their co-culture. Use of 3D mono-culture and 3D co-culture methods resulted in the modification of cell morphology and in a significant increase in ECM production. These culture methods also induced cellular changes related to EMT (epithelial-mesenchymal transition) and CAF (cancer-associated fibroblast) markers. The presented model is an easy to manufacture, well-characterized tool that can be used to study processes of the TME.

## INTRODUCTION

For decades, *in vitro* tumor models have been essential tools for understanding cancer biology and for anti-cancer agent development. Until recently, most of the *in vitro* studies employed cancer cell monolayer cultures. However, these models display significant limitations because they lack tumor-specific microenvironments [1–3]. Accordingly, major improvements are required in *in vitro* models to increase their relevance as preclinical models. First, a 3D structure should be used to enable the spatial growth of cells. It has been established that various 3D cell methods, such as spheroid, hydrogel or scaffold-based cultures, provide environmental cues more similar to those observed in physiological or pathological tissue [4–6]. Cells *in vivo* are surrounded by and interact with neighboring cells and the extracellular matrix. These reciprocal interactions are associated with the next necessary modification to *in vitro* tumor models, which is to account for the high variability of cells. Tumors are no longer considered to be masses of uncontrolled proliferating cancer cells but rather well-organized pathological organs [7] comprising various cell types, such as fibroblasts, endothelial cells, immune cells or adipocytes [8]. Accordingly, *in vitro* models require the co-culture of cells of different origins. Studies employing co-culture methods have already demonstrated and partially elucidated the mechanisms of various important biological processes such as epithelial-mesenchymal transition, metastasis, and neoangiogenesis and the transformation of fibroblasts into cancer-associated fibroblasts (CAFs) and of macrophages into tumor-associated macrophages (TAMs) [9–13]. However, the pathology of the tumor microenvironment is still not fully understood, and such an understanding is crucial for the development of new and effective cancer therapies.

In this study, we constructed a 3D breast cancer model based on a natural silk scaffold. Silk fibroin fibers have been used in medicine for decades as surgical sutures, and, recently, new applications of this biomaterial are being intensively researched, i.e., as matrices for 3D cell culture [14–16]. Owing to its biocompatibility, biodegradability and the ability to self-assemble, it has been previously successfully used in the engineering of e.g. cartilage and bone tissues [17, 18]. Recently, tumors such as hepatocarcinoma [19], mammary adenocarcinoma [20], and osteosarcoma [21] have also been successfully modeled on silk scaffolds. However, none of the above investigations have incorporated the important element of the stromal compartment of the TME.

Currently, only a few models have incorporated both the heterotypic interactions between cells and the three-dimensionality of the tissue [22–25]. We developed a breast cancer model that is based on the co-culture of cells that are most common in the tumor microenvironment: cancer cells and fibroblasts. We used the commercially available cell lines EMT6 and NIH3T3, and modified them to respectively express green and red fluorescent proteins to enable the identification of cells. To provide a 3D scaffolding system, natural silk was extracted from the cocoons of *Bombyx mori*. We optimized the methods for scaffold production, cell seeding, long-term 3D cell culture and cell detachment. We characterized our model using microscopic visualizations, cell proliferation assays, cytotoxicity assays and gene expression analyses. The use of genetic modification to produce cells that express fluorescent proteins enabled the efficient separation of cells after co-culture. This labeling was crucial for the detailed analysis of their reciprocal interactions, as studied by their gene expression patterns. The properties of the developed breast cancer model were compared with those of fibroblasts and breast cancer cells grown as a mono-culture in 3D and 2D environments.

## RESULTS

### Characterization of silk scaffolds

**Figure 1:**
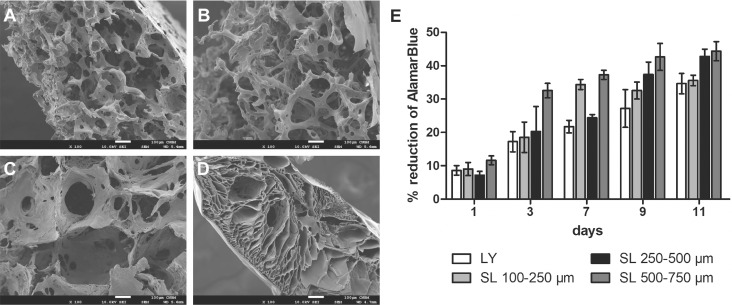
Characterization of the scaffolds prepared by various methods **(A-C)** Scanning electron microscope images of unseeded silk scaffolds prepared using the salt-leaching method with NaCl crystal sizes of (A) 100-250 μm, (B) 250-500 μm, and (C) 500-750 μm. **(D)** Scaffolds prepared by the lyophilization method. Scale bar: 100 μm. **(E)** Proliferation of EMT6 cells cultured on the scaffolds prepared by lyophilization (LY) and salt-leaching (SL) using NaCl crystals of indicated sizes, as measured by AlamarBlue assay.

To determine the optimal properties of the silk scaffold for tumor cell culture studies, four different types of scaffolds were manufactured using two methods: salt leaching and lyophilization. Scanning electron microscope analyses showed differences in scaffold thickness, pore sizes and pore shapes (Figure 1). Salt-leached scaffolds featured a uniform distribution of spherical pores, the size of which was determined by the size of porogen used (100-250 μm, 250-500 μm, or 500-750 μm) (Figure 1A–1C), whereas lyophilized scaffolds were characterized by longitudinal pores and an irregular pore structure (Figure 1D). On the bottom of the salt-leached scaffolds and on the top and bottom of the lyophilized scaffolds, thin film-like structures were observed. The cells grew mainly on the outer surface of the scaffolds prepared by lyophilization but were able to penetrate the entire structure of those prepared by salt-leaching methods (data not shown). The steadiest kinetics of cell growth were observed with the salt-leached scaffolds with a pore diameter of 250-500 μm (Figure 1E); thus, these scaffolds were selected for further experiments.

### Attachment and detachment of cells cultured on silk scaffolds

EMT6 murine breast cancer and NIH3T3 murine fibroblast cell lines were used as models in the experiments. To detect and distinguish cells, we modified them to express GFP and FP635, respectively. Following stable clone selection, the proliferation analyses confirmed that cell modifications had no effect on the cell growth kinetics (Supplementary Figure 1). Cells of both lines attached well to the scaffolds with no significant differences 5 h after seeding (Supplementary Figure 2A). However, fibroblasts attached to the silk scaffolds faster than cancer cells.

To detach cells from the scaffolds for further analyses, such as fluorescence cytometry, cell sorting or RNA isolation, several detachment conditions were tested. The best results were obtained using a mixture of collagenase and dispase solution for 90 min at 37°C (Supplementary Figure 2B, 2C). These conditions resulted in the highest number of viable, single cell suspensions, whereas the other conditions led to higher cell mortality (Supplementary Figure 2C).

### The morphology of cells cultured in 3D conditions

**Figure 2:**
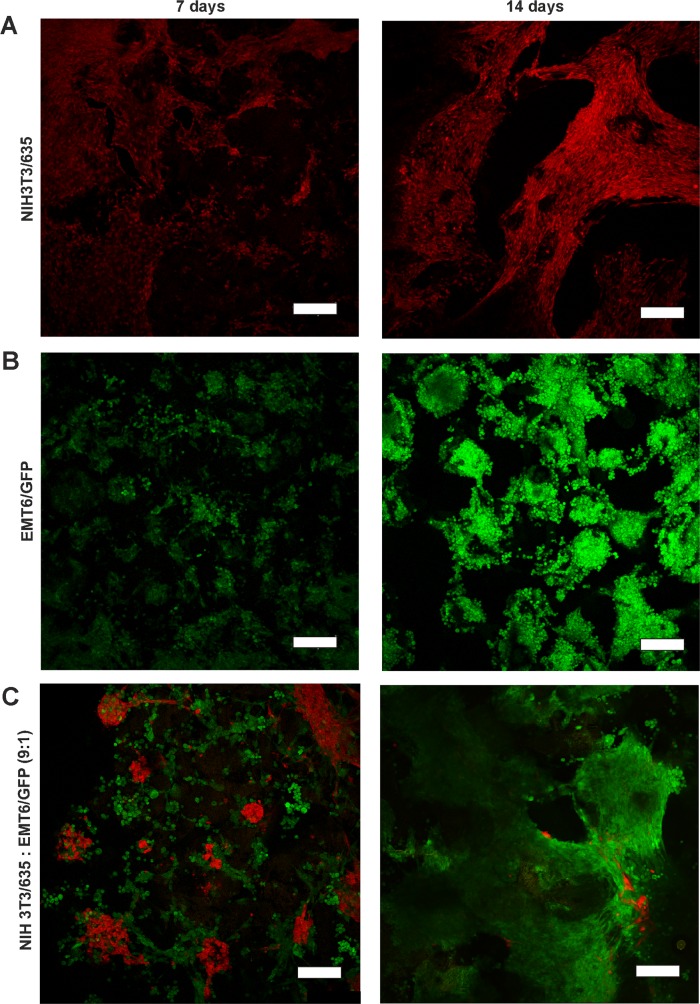
CLSM images of NIH3T3/635 fibroblasts (red) and EMT6/GFP cancer cells (green) after 7 (left) and 14 (right) days of culture **(A)** NIH3T3/635 and **(B)** EMT6/GFP cells were cultured as mono-cultures, and **(C)** as co-cultures of fibroblasts and cancer cells seeded at a 9:1 ratio. Scale bar: 200 μm.

**Figure 3:**
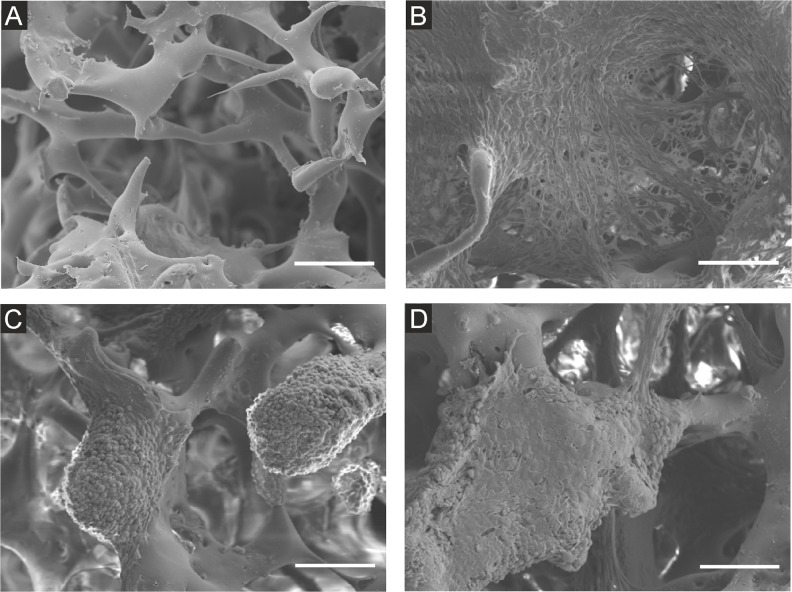
Scanning electron microscope images of NIH3T3/635 and EMT6/GFP cells cultured on the silk scaffolds for 14 days **(A)** Unseeded scaffold, **(B)** NIH3T3/635 mono-culture, **(C)** EMT6/GFP mono-culture, **(D)** co-cultures of NIH3T3/635 and EMT6/GFP cells seeded at a 9:1 ratio. Scale bar: 100 μm.

Cells growing on the scaffolds were visualized by CLSM (Figure 2, Supplementary Figure 3) and scanning electron microscopy (Figure 3) after 7 and 14 days of culture. In mono-cultures, both fibroblasts and cancer cells attached and spread successfully on the scaffolds. The morphology of EMT6 cells was mostly rounded (Figure 3C), whereas fibroblasts showed spindle-shaped, elongated cell bodies (Figure 3B). Fibroblasts grew spatially, spread across the pores and formed sheet-like structures (Figure 2A, 3B). Breast cancer cells grew in tight, round groups, forming spheroidal structures (Figure 2B, 3C). Based on the microscopic images in all culture types, cells grew preferentially on the outer surface of the scaffolds, with a smaller number of cells in the core of the scaffold (data not shown).

In co-culture studies, breast cancer cells overgrew fibroblasts regardless of the initial ratio of seeded cells (Figure 2C, Supplementary Figure 3). If the fibroblasts to breast cancer cell seeding ratio was 9:1, after two weeks of co-culture, only a few fibroblasts could be detected. Moreover, compared to fibroblast mono-cultures, the singular cells of the growing mass of cancer cell mono-cultures could be more easily distinguished, as indicated by the scanning electron microscope images (Figure 3B, 3C). However, when cancer cells were co-cultured with fibroblasts, it was difficult to discriminate separate cells (Figure 3D) even though after 14 days of culture only a few fibroblasts were present (Figure 2C).

### Proliferation of breast cancer cells and fibroblasts in mono- and co-cultures on the silk scaffold

**Figure 4:**
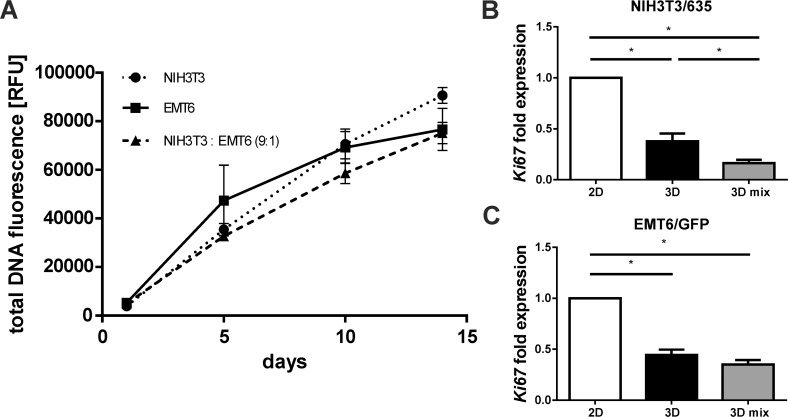
Proliferation of cells cultured on the silk scaffolds in mono- and co-culture **(A)** NIH3T3 and EMT6 cell overall proliferation measured at day 1, 5, 10 and 14 by quantification of total DNA using QuantiFluor. Results represent the means of three independent experiments in triplicate; error bars represent the SEMs. **(B, C)** Relative expression of cell proliferation marker *ki67* in (B) NIH3T3/635 and (C) EMT6/GFP cells mono-cultured in 2D and 3D cultures and in a co-culture on 3D silk scaffolds at a 9:1 ratio, as measured by real-time PCR analyses. The experiment was repeated at least three times; error bars represent the SEMs. ^*^ indicates p < 0.05.

Cell proliferation on the silk scaffold was measured indirectly based on cell metabolic activity using the AlamarBlue assay (Supplementary Figure 4) and directly by total DNA quantification (Figure 4A). Cancer cells as well as fibroblasts proliferated slower in 3D than in 2D culture (Supplementary Figure 4A, 4B). Both proliferation assays showed steady growth of cells on the silk scaffolds for a period of two weeks (Figure 4A). Cells remained viable on the scaffold after 30 days of culture (data not shown). Consistent with metabolic assays, DNA quantification results showed the trend of slower proliferation of cells when co-cultured (Figure 4A). Real-time PCR analyses were performed to assess the expression levels of the *ki67* proliferation marker in cells cultured in 2D and 3D mono-cultures and 3D co-cultures. Analyses confirmed lower expression of *ki67* in cells cultured in 3D compared with cells from 2D culture (Figure 4B, 4C). Observed differences were statistically significant. Moreover, the expression of *ki67* was significantly lower in fibroblasts co-cultured in 3D than in those from 3D mono-culture (Figure 4B).

### Analysis of the growth kinetics of cells in mono- and co-culture

As noted above, regardless of the initial ratio of seeded cells in co-cultures, cancer cells overgrew fibroblasts (Figure 2, Supplementary Figure 3). The additional flow cytometry analyses confirmed the microscopic observations. Analyses of the green and red fluorescence of modified cells demonstrated that the fibroblast number dramatically decreased during the progress of co-culture (Table 1). The 9:1 ratio of seeded fibroblasts to cancer cells changed to approximately 1:4 after 12 days of co-culture.

**Table 1:** Ratio (%) of cells of a particular type on the silk scaffolds after 8, 10 and 12 days of co-culture depending on the ratio of seeding, as measured by flow cytometry

Seeding ratio (%)	8^th^ day		10^th^ day		12^th^ day	
**NIH3T3/635 :**	**NIH3T3/635**	**EMT6/GFP**	**NIH3T3/635**	**EMT6/GFP**	**NIH3T3/635**	**EMT6/GFP**
**EMT6/GFP**						
**90:10**	34.9 ± 15.6 %	65.1 ± 15.6 %	27.2 ± 15.6 %	72.9 ± 15.6 %	16.9 ± 10.5 %	83.1 ± 10.5 %
**50:50**	7.5 ± 2.6 %	92.7 ± 2.6 %	2.9 ± 0.9 %	97.1 ± 0.9 %	2.4 ± 0.6 %	97.6 ± 0.6 %
**10:90**	1.9 ± 0.5 %	98.1 ± 0.5 %	1.6 ± 0.7 %	98.4 ± 0.7 %	1.4 ± 0.8 %	98.6 ± 0.8 %

The experiment was repeated three times; results represent the means ± SDs.

**Figure 5:**
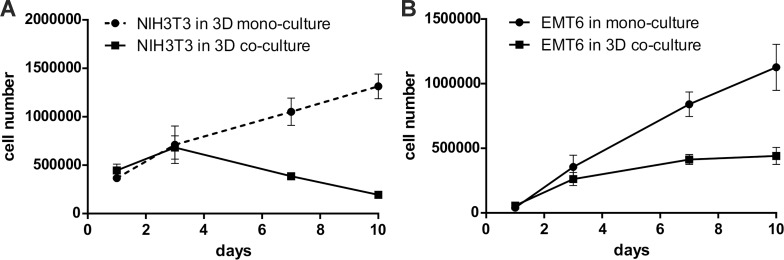
Growth kinetics of NIH3T3/635 and EMT6/GFP cells seeded in equal quantities on the silk scaffolds in mono- and co-culture, as measured by counting the number of red or green fluorescent cells using a Fuchs-Rosenthal counting chamber and a fluorescence microscope **(A)** For the experiment, 4.5 × 10^5^ fibroblasts were seeded onto the scaffolds as a mono-culture or together with 0.5 × 10^5^ cancer cells (9:1 ratio). The red cells (NIH3T3/635) were counted after detachment on days 1, 3, 7, and 10. **(B)** For the experiment, 0.5 × 10^5^ cancer cells were seeded onto the scaffolds alone or co-cultured with 4.5 × 10^5^ fibroblasts. The number of cancer cells was determined as above. The experiments were repeated three times; results are presented as the means ± SEMs.

In another set of experiments, cells were seeded in the same quantity in mono- and co-cultures. Growth kinetics of 4.5 × 10^5^ NIH3T3/635 cells seeded in mono-culture were compared with 4.5 × 10^5^ NIH3T3/635 cells co-cultured with 0.5 × 10^5^ EMT6/GFP cells. Cell counts showed a decrease in fibroblasts after the third day of co-culture, whereas in the mono-culture it gradually increased (Figure 5A). To determine the cancer cell proliferation rate in mono- and co-cultures, 0.5 × 10^5^ EMT6/GFP cells were seeded alone and in co-culture with 4.5 × 10^5^ NIH3T3/635 cells, cultured and then counted. The results showed that cancer cells proliferated considerably faster when cultured alone (Figure 5B). Supplementary Figure 5 shows a 10-day graphic representation of the changes in percentages of both cell types during the co-culture of fibroblasts and cancer cells seeded at a 9:1 ratio, respectively.

Due to observed differences in the number of cells of a particular type during co-culture, the ratio of seeded cells was established at 9:1 of fibroblasts to cancer cells, and this model was applied for further studies.

### Cytotoxicity of doxorubicin in 3D cultures

**Figure 6:**
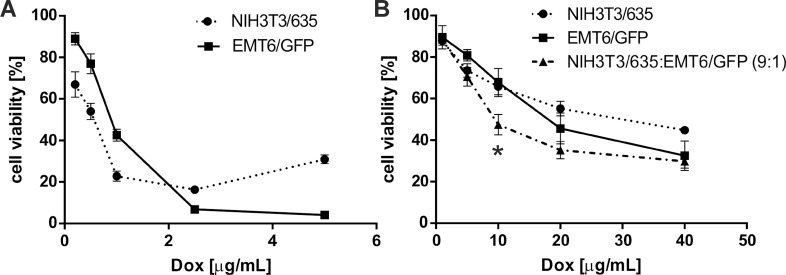
Toxicity of Dox on NIH3T3/635 and EMT6/GFP cells in **(A)** 2D culture and **(B)** 3D culture on silk scaffolds as measured by AlamarBlue assay. (A) In 2D experiments, Dox was added 24 h after seeding of the cells. (B) In 3D experiments, Dox was added 10 days after seeding of cells. Cell viability was measured 48 h after the addition of Dox and compared with non-treated control cells. The results are expressed as the means of at least three independent experiments, each in triplicate; error bars represent the SEMs. ^*^ indicates p < 0.05.

Both tumor cells and fibroblasts cultured in 3D conditions were considerably more resistant to doxorubicin (Dox) than those cultured in standard 2D monolayers (Figure 6). At a Dox dose of 1 mg/mL, the mortality of EMT6/GFP and NIH3T3/635 cells in 2D was approximately 60% and 80%, respectively (Figure 6A). To achieve the same degree of Dox toxicity in 3D culture, the dose had to be increased 40-fold (Figure 6B). In co-culture on 3D silk scaffolds, cells displayed a slight trend toward higher sensitivity to Dox than in mono-culture, whereas at a dose of 10 μg/mL the differences were statistically significant.

### Gene expression analyses – analysis of the breast cancer cells cultured in mono- and co-culture

To study the influence of using 3D culture conditions on breast cancer cells and fibroblasts, we analyzed the expression of selected genes in these cells that are important for tumor development and progression. Gene expression was studied in 3D co-cultured cells and in mono-cultured cells in 2D and 3D models. After 7 days of co-culture, cells were harvested and sorted based on their fluorescence. Then, RNA was isolated from corresponding cells.

**Figure 7:**
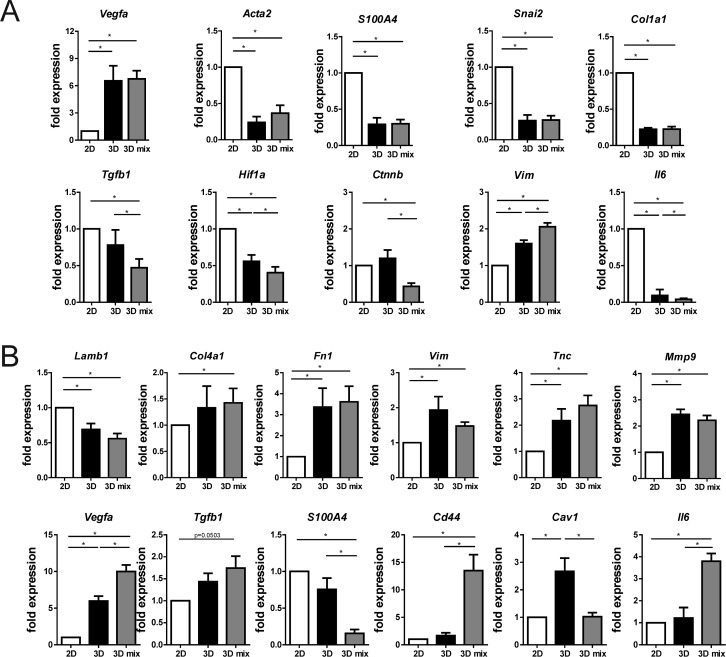
Effect of using the 3D co-culture model on the expression level of selected genes in **(A)** cancer cells and **(B)** fibroblasts as assessed by real-time PCR. Standard 2D mono-cultures (2D), mono-cultures on silk scaffolds (3D), and co-cultures of cancer cells with fibroblasts on 3D silk scaffolds (3D mix) were compared. Expression levels of analyzed genes were normalized to β-tubulin expression levels. Experiments were repeated at least three times in triplicate. Graphs represent mean fold changes ± SEMs. ^*^ indicates p < 0.05.

EMT6/GFP cells expressed genes characteristic of the epithelial-mesenchymal transition (EMT) phenotype at a significantly lower level when cultured in 3D than in 2D conditions (Figure 7A). These differences included a significant decrease of *Acta2*, *Snai2*, *S100a4* and *Col1a1* gene expression. Co-culture with fibroblasts in 3D did not modify the expression levels of these genes in EMT6/GFP cells. Additionally, mRNA of *Vegfa* was significantly altered in both 3D mono- and co-culture conditions in cancer cells, with an approximately 6-fold increase in *Vegfa* expression, compared with the expression of this gene in 2D cultured EMT6/GFP cells (Figure 7A). Furthermore, we found that genes encoding proteins such as TGF-β1, HIF-1α and β-catenin were downregulated in EMT6/GFP cells co-cultured with fibroblasts in 3D compared with cells in 3D mono-culture (Figure 7A). Additionally, mRNA of interleukin 6 (*Il6*) was significantly altered in 3D mono-culture (with an approximately 10-fold decrease compared with the expression of *Il6* in 2D cultured EMT6/GFP cells) and after co-culture with fibroblast (significant decrease comparing with 3D mono-cultured cancer cells). Moreover, these cells were characterized by a significantly higher vimentin expression level compared with respective 3D control cells (Figure 7A).

### Gene expression analyses - analysis of the fibroblast cells cultured in mono- and co-culture

Upon 3D mono- and co-culture, fibroblasts were found to express significantly higher levels of genes associated with extracellular matrix production and remodeling compared with the expression in cells grown in 2D conditions (Figure 7B). We observed a significant increase in the quantity of tenascin C, fibronectin 1, collagen IV and matrix metallopeptidase 9 mRNAs in NIH3T3/635 cells on 3D silk scaffolds compared to fibroblasts in 2D culture. Moreover, the results showed a significant increase in the vimentin expression level and a downregulation of laminin B1 mRNA in fibroblasts from both 3D cultures compared with NIH3T3/635 cells from 2D culture (Figure 7B). Upon co-culture with tumor cells, fibroblasts downregulate the expression of the CAF marker *S100a4* compared with their 3D mono-cultured counterparts (Figure 7B). However, the expression of other CAF markers, such as *Cd44* and *Vegfa*, was significantly upregulated (Figure 7B). Furthermore, in NIH3T3/635 cells after 3D co-culture with cancer cells, a trend of increasing expression was found with *Tgfb1* and significantly decreased levels of caveolin-1 mRNA were observed. Additional analyses showed a significant increase in *Il6* mRNA levels in co-cultured fibroblasts compared with their 3D mono-cultured counterparts (Figure 7B).

## DISCUSSION

The limitations of current standard models in their ability to assess the efficacy of anti-cancer agents and various cell interactions in the tumor microenvironment requires the development of novel, advanced *in vitro* tumor models. The presented model incorporated three-dimensional *in vitro* culture as a tool to bridge the gap between standard 2D *in vitro* models and preclinical mouse models. The model enabled spatial cell growth on a porous, ECM-like scaffolding structure, which mimicked the *in vivo* tissue environment and facilitated the maintenance of appropriate cell physiology. The most important characteristic of our model was the simultaneous incorporation of two types of cells, which formed a “tumor organ”. This application of heterotypic co-culture enabled the analysis of the direct, reciprocal interactions between cancer cells and surrounding stromal cells.

The biomaterial used for the construction of the model – natural silk fibroin – is relatively inexpensive, easy to obtain, non-toxic and, following the removal of sericin layer, no activating adaptive immune response [26]. Our initial investigations focused on selecting the best scaffold technology for the culture of tumor cells. We demonstrated that silk scaffolds with a pore size of 250-500 μm enabled the best cell infiltration and proliferation. Thus, these scaffolds were selected for further studies and model development.

In the presented model, we applied two commercially available, well-characterized, established cell lines from the same inbred mouse strain Balb/c: fibroblasts NIH3T3 and breast cancer cells EMT6. According to previous research, no significant differences in expression of genes related to ECM production were observed between primary lung fibroblasts co-cultured with primary lung cancer cells and the established lung fibroblast cell line CCL-210 co-cultured with A549 cancer cells [27]. Thus, the established cell lines can be used for reconstruction of the natural environment and can also allow for high study reproducibility.

Given the increasing importance of the tumor microenvironment (TME) in cancer biology, numerous *in vitro* co-culture models have been developed [9–13, 22–25]. Still, very few of these models enable the study of the direct and reciprocal cell interactions between stromal and tumor cells. Some models use indirect co-culture, either based on cells grown in separated compartments or with the use of conditioned media (CM) from particular type of cells [9, 28]. These methods allow only for studying the effects of paracrine signaling but not direct cell-cell interactions, and by using CM, it is possible to capture one sided cellular processes but not reciprocal interactions between both cell types. We developed the system that enable to study the direct cell-cell interactions. In order to perform these analyses, first we optimized a method of enzymatic cell detachment to harvest cells from the 3D culture. Moreover, we resolved a problem of the separation of each cell type from a heterotypic co-culture. Previously, the identification of cells was based on the recognition of the cell specific surface antigens by antibodies using flow cytometry or magnetic-activated cell sorting (MACS) methods. However, finding markers exclusive for one population of cells might be challenging, particularly taking into consideration changes in the gene expression profiles upon heterotypic co-culture. The genetic modifications of NIH3T3 and EMT6 cells enabled sorting them using flow cytometry what guaranteed close to 100% purity of the isolated cell populations.

Both cell types attached and proliferated well on the scaffolds, confirming previous reports that silk fibroin is a superior biomaterial for supporting cell culture [29–31]. We observed differences in morphology, the proliferation rate, and sensitivity to drug between cells cultured in 2D and those in 3D and between cells cultured in 3D mono-cultures and those in co-cultures. Fibroblasts in a mono-culture on 3D silk scaffolds exhibited elongated morphology and formed net-like structures spreading over the pores of the scaffold similarly as in other 3D systems: collagen- [32] and electrospun aligned PLA scaffold-based cultures [33]. Interestingly, when fibroblasts were added to the co-culture model they participated in the formation of spheroid structures. Moreover, in the co-culture model, it was difficult to discriminate singular cells by scanning electron microscopy images. A possible explanation for this phenomenon might be related to the embedding of cells into the thick layer of extracellular matrix produced by cells in these conditions.

The proliferation rate of cells on 3D silk scaffolds was lover compared with those in 2D cultures. These observations were in agreement with previous studies [34, 35] and were reported to be more similar to those observed in tumors *in vivo*. Moreover, we observed different growth kinetics in both cell types upon 3D co-culture. In a study by X. Wang et al., non-cancerous breast epithelial cells were also found to proliferate slower in the presence of stromal cells in 3D culture [36]. Additionally, in our co-culture model the number of fibroblasts decreased during culture. We hypothesize that cancer-induced autophagy of fibroblasts might be the reason for the observed effect. It was indicated that the activation of the TGF-β pathway in stromal cells induced their metabolic reprogramming resulting in increased autophagy/mitophagy and downregulation of CAV-1 [37]. Our initial results indicated a significantly lower expression level of *Cav1* and a higher expression level of *Tgfb1* in fibroblasts in 3D co-culture compared with fibroblasts in 3D mono-culture. However, determining whether these factors were responsible for the reduced number of fibroblasts requires further research, particularly regarding the amount and the activity of the corresponding proteins.

The *in vitro* 3D cancer model seems to be the model of choice for testing the abilities of a drug to penetrate the tumor; that is, it can be used to determine the effective dose of a drug and to study tumor biology in response to the drug. Our research showed that cells grown in 3D culture were significantly more resistant to the cytotoxic effect of Dox than those grown in 2D. A similar effect has been reported for Dox and paclitaxel in other 3D cancer models [38, 39].

To further investigate the processes occurring in fibroblasts and cancer cells upon transferring them into 3D mono- and co-culture, we separately analyzed the gene expression patterns in both types of cells. When cultured on 3D silk scaffolds, EMT6 cancer cells. were characterized by a more epithelial phenotype than the cells in standard 2D culture. These phenotypic conclusions are based on the cell morphology and the expression levels of selected genes characteristic for EMT reviewed elsewhere [40]. The expression levels of most of these genes did not change after the addition of fibroblasts to the co-culture; however, a significant downregulation of β-catenin and an increased expression of vimentin were observed, together with a trend of increased α-SMA expression. In MDA-MB-231 breast cancer cells, knockdown of β -catenin led to an increase in cell mobility and in mesenchymal vimentin expression, which suggested an EMT [41]. The transition of EMT6 cells into a 3D environment resulted in the manifestation of their more epithelial phenotype. However, during co-culture, their phenotype further transitioned toward a mesenchymal one.

Transferring fibroblasts onto 3D silk scaffolds resulted in changes in their gene expression profiles, especially in those genes responsible for extracellular matrix production and remodeling. It has been shown before that 3D culture provides stromal cells with environmental cues needed for maintaining their physiology [42]. The cancer cells in co-culture did not significantly influence the expression of these genes in fibroblasts.

Moreover, we assessed the expression level of genes that are acknowledged to be involved in the transition of fibroblasts into CAFs. CAFs are a complex and heterogeneous cell population, and their molecular definition is still under debate [43]. In our model, fibroblasts were characterized by a significant decrease in *S100a4* and a significant increase in *Cd44* expression following co-culture with tumor cells in 3D. Overexpression of CD44, a glycoprotein on the surface of mesenchymal cells in the TME, has been shown to lead to acquisition of the CAF phenotype [44] and to support stemness and drug resistance in tumors [45]. Additionally, we found that NIH3T3/635 cells showed a downregulated caveolin-1 mRNA level in the presence of tumor cells compared with those in mono-culture. Martinez-Outschoorn et al. showed that loss of CAV-1 was a critical initiating factor for CAF transformation in stromal fibroblasts [46]. These data plus a significant increase in *Vegfa* expression with a trend of increased *Tgfb1* and *Acta2* expression (data not shown) in fibroblasts upon co-culture with cancer cells indicated that their transformation into CAFs was initiated.

Furthermore, the interplay between cancer cells and fibroblasts in terms of interleukin 6 expression was observed. The downregulation of *Il6* mRNA levels in EMT6 cells in 3D mono- and co-culture was coupled with simultaneous upregulation of this cytokine in 3D co-cultured fibroblasts. A similar effect was seen in the mRNA levels of the main EMT driver *Tgfb1*. We observed an increase in the expression of *Vegfa* in cancer cells that appeared to be independent of *Hif1a* expression [47]. However, HIF-1a is regulated mostly at the protein level [48]. To understand the processes underlying these changes, further studies are needed at the protein level.

## MATERIALS AND METHODS

### Silk fibroin extraction

*Bombyx mori* silkworm cocoons were obtained from the Institute of Natural Fibers and Medicinal Plants, Poznan, Poland. Silk fibroin was extracted as previously described by Rockwood DN et al. [49]. Briefly, 5 g of cocoons were cut into pieces using scissors, and 4.24 g sodium carbonate (Sigma, St. Louis, MO) was dissolved in 2 L distilled boiling water. Cocoon pieces were boiled for 30 min with gentle stirring to remove sericins. Next, silk fibroin was washed three times in 2 L distilled water for 20 min and dried overnight in a fume hood. After drying, silk fibroin was dissolved in 9.3 M lithium bromide (Sigma, St. Louis, MO) for 4 h at 60°C, transferred to a ZelluTrans dialysis tube with an MWCO of 3500 kDa (Carl Roth, Karlsruhe, Germany) and dialyzed against distilled water. Distilled water was changed six times over 48 h. Next, the silk fibroin solution was centrifuged twice for 20 min at 5000 × g. The concentration of silk was determined gravimetrically using the following formula: ((W1-Wc)/(W2-Wc))×100%, where W1 is the weight of 1 mL silk solution in the weighing container, W2 is the weight of the dry silk film in the weighing container after 24 h in a fume hood, and Wc is the weight of the weighing container.

### Silk fibroin scaffold preparation

Porous scaffolds were manufactured using a salt leaching technique [49]. Sodium chloride (Thermo Fisher Scientific Inc., Waltham, MA) of a defined particle size was obtained by sieving through 100 μm, 250 μm, 500 μm and 750 μm test sieves (Sigma, St. Louis, MO; Retsch Technology, Haan, Germany). Round polyethylene (PE) containers 2 cm in diameter were filled with 0.5 mL silk solution (approximately 8% wt), then 1 g salt particles was added, and containers were placed at 60°C for a minimum of 5 days. After incubation, containers were immersed in distilled water for 2 days to leach out the salt. Next, scaffolds were cut into disks with a diameter of 6 mm and a height of 1.5 mm using a biopsy punch (PFM Medical, Koln, Germany). Scaffolds were washed 3 times with 70% ethanol (POCH, Gliwice, Poland). After an additional three washes in PBS (Sigma, St. Louis, MO), scaffolds were immersed in complete cell culture medium and incubated for 24 h before seeding of cells. For lyophilized scaffolds, a 3% wt silk solution was frozen in PE containers and lyophilized (Labconco, Kansas City, MO). Scaffolds where further prepared for cell culture as described above.

### Cell culture

The original cell lines were obtained from American Type Culture Collection (ATCC, Manassas, VA, USA) and routinely tested for mycoplasma contamination using VenorGem Mycoplasma Detection Kit (Minerva Biolabs GmbH; Berlin Germany). NIH3T3 mouse fibroblasts, EMT6 mouse breast cancer cell lines and HEK293T cells were maintained in complete medium composed of Dulbecco's Modified Eagle's Medium (DMEM; PAA Laboratories GmbH, Pasching, Austria) supplemented with 10% fetal bovine serum (PAA Laboratories GmbH, Pasching, Austria) and 80 μg/mL gentamycin (KRK, Novo Mesto, Slovenia). Cells were grown using standard culture conditions (37°C, humidified atmosphere containing 5% CO_2_).

### Modification of cells

The lentiviral vectors Lv-FP635 and Lv-GFP were used for the expression of far red fluorescence protein (turboFP635) and the expression of green fluorescence protein (GFP), respectively. The vectors were produced by co-transfection of HEK293T cells with three plasmids: pMD2.G, p8.91, and pWPXL-FP635 or pWPXL-GFP (Addgene, Cambridge, MA). Lentivirus containing medium was collected after 48 h, filtered with 0.2 μm filters, aliquoted and stored at -80°C until further use. For the experiments, 1 × 10^5^ NIH3T3 and EMT6 cells were transduced using 1 mL lentivirus containing medium in the presence of 5 μg/mL polybrene (Sigma, St. Louis, MO). Stable clones of NIH3T3/635 and EMT6/GFP cells were obtained by clonal selection and analyzed by flow cytometry and proliferation assays.

### Cell culture on silk scaffolds – three-dimensional (3D) culture

Unless indicated otherwise, 3 × 10^5^ cells were suspended in 20 μL of complete cell culture medium and seeded on the top of the scaffolds in a 24-well non-treated TCP (Nunc, Roskilde, Denmark). After 1 h of incubation, 2 mL complete medium was added and cells were cultured for the indicated periods of time. During culture, medium was changed every two days. Scaffolds with cells were transferred to 6-well plates for long-term culture and supplemented with 10 mL culture medium. Fibroblasts and breast cancer cells were seeded as mono-culture and co-culture at 1:1, 1:9 and 9:1 ratios.

### Cell attachment analysis

Silk scaffolds were seeded with 3 × 10^5^ NIH3T3/635 or EMT6/GFP cells as described above. Cells adherence to scaffolds was assessed by counting non-attached cells after 1 and 5 h of incubation. Cells were counted using a hemocytometer (Fuchs-Rosenthal counting chamber). The experiment was repeated three times.

### Cell detachment analysis

Accutase solution (Sigma, St. Louis, MO) and a mixture of collagenase/dispase solution (Roche, Basel, Switzerland) were used to test for enzymatic detachment of cells from the silk scaffolds. NIH3T3/635 or EMT6/GFP cells were seeded onto the scaffolds and cultured for 48 h in mono-culture and in co-culture at a 1:1 ratio. Scaffolds with cells were washed with PBS and 1 mL of detaching solution (Accutase 1X or collagenase (0.1 U/mL)/dispase (0.8 U/mL)) was added per well and incubated at 37°C as indicated. Quantity and viability of detached cells was assessed by hemocytometric counting using trypan blue (Sigma, St. Louis, MO). The experiment was repeated three times.

### Cell proliferation assays

Cell proliferation on 3D silk scaffolds was measured by total DNA quantification using a QuantiFluor dsDNA system (Promega, Madison, WI) according to the manufacturer's protocol. Briefly, 5 × 10^4^ unmodified NIH3T3 cells, EMT6 cells, or a mix of both cell lines at a 9:1 ratio was seeded onto the scaffolds. At days 1, 5, 10 or 14, the cells on the scaffolds were washed with PBS and lysed in 750 μL Cell Lytic M reagent (Sigma, St. Louis, MO) for 1 h with shaking. Lysates were frozen at -20°C. For the assay, lysates were diluted 10 × with Cell Lytic Reagent M and mixed at a 1:1 ratio with the supplied working solution of dsDNA dye. After a 5 min incubation at RT, fluorescence was measured using a Victor X3 Multimode Plate Reader (PerkinElmer, Waltham, MA) controlled by the PerkinElmer 2030 Workstation software (PerkinElmer, Waltham, MA). The excitation wavelength of 504 nm and emission of 531 nm were used. The experiment was repeated three times in triplicate.

Cell proliferation based on metabolic activity was measured using AlamarBlue reagent (Bio-Rad AbD Serotec, Kidlington, UK), according to the manufacturer's protocol. Briefly, 5 × 10^4^ of indicated cells were seeded onto the scaffolds. Every 2-3 days, scaffolds with cells were transferred to a fresh 48-well plate (Nunc, Roskilde, Denmark) and supplemented with 1 mL complete cell culture medium containing 10% AlamarBlue reagent. After 3 h of incubation, 100 μL medium from each well was transferred to a fresh, black 96-well plate (Nunc, Roskilde, Denmark) and fluorescence was measured at the excitation wavelength of 560 nm and emission wavelength of 590 nm using a Victor X3 Multimode Plate Reader (PerkinElmer, Waltham, MA) controlled by the PerkinElmer 2030 Workstation software (PerkinElmer, Waltham, MA). The experiments were repeated at least three times.

### Scanning electron microscopy

For the experiments, 3 × 10^5^ NIH3T3/635 and EMT6/GFP cells were seeded onto the scaffolds in mono-cultures and in co-cultures at the ratios of 1:1, 9:1 or 1:9. After 14 days of culture, cells on the scaffolds were fixed using 2.5% glutaraldehyde (Sigma, St. Louis, MO) in PBS for 30 min, washed three times in PBS and dehydrated by immersion for 15 min successively in 50%, 70%, 85%, 95%, and 100% ethanol (POCH, Gliwice, Poland). Next, scaffolds with cells were dried overnight in a fume hood and sputter-coated with AuPd under a vacuum in a Quorum Sputter Coater Q150T ES (Quorum Technologies, Ringmer, UK). Cells were visualized using a JSM 5900 LV scanning electron microscope (JEOL Ltd, Japan) at 10 kV.

### Confocal laser scanning microscopy (CLSM)

Scaffolds were seeded with 3 × 10^5^ NIH3T3/635 or EMT6/GFP cells in mono-cultures and co-cultures at the ratios of 1:1, 9:1 or 1:9. After 7 or 14 days of culture, scaffolds with cells were transferred into LabTek chambered cover glasses (Nunc, Naperville, IL). Cells were visualized live in culture medium using a Leica TCS SP5 X confocal laser scanning microscope (Leica, Wetzlar, Germany) under a 4X objective controlled by the Leica Application Suite Advanced Fluorescence (LAS AF) Lite software (Leica, Wetzlar, Germany). Images were z-stacks of 200 μm scans. Cells were visualized using a white light laser (WLL) at an excitation wavelength of 488 nm and emission bandwidth of 500-551 nm for GFP and an excitation wavelength of 588 nm and emission bandwidth of 613-670 nm for turboFP635.

### Flow cytometry analysis

Scaffolds were seeded with 3 × 10^5^ NIH3T3/635 and EMT6/GFP cells at 1:1, 9:1 and 1:9 ratios. After 8, 10 or 12 days of co-culture, cells were detached from the scaffolds using collagenase/dispase solution at 37°C for 90 min as described above. Detached cells were washed three times in PBS and analyzed using a FACSAria flow cytometer (BD Biosciences Pharmingen, San Jose, CA) and FACSDiva v6.1.2 software (BD Biosciences Pharmingen, San Jose, CA). The green fluorescence from GFP and red fluorescence from turboFP635 were collected using 530/30 nm and 695/40 nm bandpass filters, respectively. For excitation of both fluorescent proteins 488 nm blue laser was employed. Percentages of green and red fluorescent cells were quantified for each time point. The experiments were repeated at least three times.

### Cytotoxicity assay

The cytotoxicity assay was performed using 2D and 3D cell culture conditions. For 2D cell culture, 2.5 × 10^4^ NIH3T3/635 or EMT6/GFP cells were seeded into a 96-well plate. The following day, doxorubicin (Dox; Adriamycin, Pfizer Inc., New York City, NY) was added at the following final concentrations: 0.2, 0.5, 1, 2.5 or 5 μg/mL. For 3D cell cultures, 3 × 10^5^ NIH3T3/635 cells, EMT6/GFP cells or a mix of both cell lines at a 9:1 ratio, were seeded onto the scaffold. Doxorubicin was added at day 10 at the following concentrations: 1, 5, 10, 20 or 40 μg/mL. The cytotoxic effect of doxorubicin on cells from both 2D and 3D cultures was measured after 48 h using AlamarBlue reagent according to the manufacturer's protocol, as indicated above. The percentage of viable cells was calculated based on the fluorescence of un-treated controls for the 2D and 3D cultures. The experiment was repeated at least three times.

### Analysis of kinetics of cell growth on 3D scaffolds

Silk scaffolds were seeded with 0.5 × 10^5^ EMT6/GFP cells, 4.5 × 10^5^ NIH3T3/635 cells or a mix of 5 × 10^5^ NIH3T3/635 and EMT6/GFP cells at a 9:1 ratio. After 1, 3, 7 or 10 days of culture, cells were detached and the cell number and cell viability were evaluated by hemocytometer counting using trypan blue. The percentages of red and green fluorescent cells in the mixed culture were calculated by flow cytometric analysis.

### Cell sorting

Samples were analyzed using BD FACS Aria™III (Becton Dickinson) flow cytometer (cell sorter). The instrument setup (optical alignment), stability and performance test was performed using CST system (Cytometer Setup and Tracking) from Becton Dickinson company. FACSFlow solution (Becton Dickinson) was used as sheath fluid. The configuration of flow cytometer was as follows: 100 μm nozzle and 20 psi (0,138 MPa) sheath fluid pressure. The cells were characterized by two non-fluorescent parameters: forward scatter (FSC) and side scatter (SSC), and two fluorescent parameters: green fluorescence from GFP collected using 530/30 bandpass filter (502 long pass filter, FITC-A detector) and red fluorescence from turboFP635 collected using 695/40 bandpass filter (655 long pass filter, PerCP-Cy5.5 detector). For excitation of both fluorescent proteins 488 nm blue laser was employed. The flow cytometry analyses were performed by using logarithmic gains and specific detectors settings. The threshold was set on the FSC signals. Data were acquired in a four-decade logarithmic scale as area signals (FSC-A, SSC-A, FITC-A and PerCP-Cy5.5-A) and analyzed with FACS DIVA software (Becton Dickinson). Cellular populations were defined by gating in the dot plots of green fluorescence (FITC-A) versus red fluorescence (PerCP-Cy5.5-A). Each sample was analyzed in triplicates. Sort regions were then defined on bivariate dot plot (FITC-A vs. PerCP-Cy5.5-A) that delineated distinct populations. Cell sorting preceded doublets discrimination procedure with the use of height versus width scatter signals measurement, to discriminate single cells from conglomerates allowing high purity sort. The FACS Aria™III cell sorter settings were established for gaining highest purity level (4-way purity was selected from Sort Layout window). The cells were sorted into 5 ml cytometric tubes with the culture medium.

### RNA isolation and reverse transcription

NIH3T3/635 and EMT6/GFP cells mono-cultured in 2D and 3D conditions were detached as mentioned previously, then RNA was isolated from cells using TRI reagent (Sigma, St. Louis, MO) according to the manufacturer's protocol. Co-cultured cells were subjected to cell sorting before RNA isolation. For real-time PCR analyses, cDNA matrices were obtained by reverse transcription of RNA samples using an iScript cDNA synthesis kit (BioRad, Hercules, CA) according to the manufacturer's protocol.

### Real-time polymerase chain reaction

For specific detection, hydrolytic probes and primers were designed using the Universal Probes Library (Roche, Basel, Switzerland). Probes were acquired from Roche (Basel, Switzerland), and primers were purchased from Sigma (St. Louis, CA). A list of primers and corresponding probes can be found in Supplementary Table 1. Sequence-specific amplification with real-time PCR was performed using a Probes Master kit (Roche, Basel, Switzerland) on the LightCycler 480 (Roche, Basel, Switzerland). Gene expression was normalized to β-tubulin expression for each sample. Relative gene expression was calculated using the ΔΔCt method. The experiments were repeated at least three times in triplicate.

### Statistical analysis

To determine statistical significance, analyses were performed using GraphPad Prism v5.01 software (GraphPad Software, La Jolla, CA). Data were analyzed by Student's *t*-test when comparing two groups or with a two-way analysis of variance (ANOVA) with Bonferroni's multiple comparison test when comparing more than two groups. The differences between groups were considered significant at a p < 0.05.

## CONCLUSIONS

We generated and characterized an advanced *in vitro* 3D breast cancer model to study tumor biology and the effectiveness of anti-cancer agents. The 3D cancer model was built on the basis of the simultaneous co-culture of two types of cells (breast cancer cells and fibroblasts) on a natural silk scaffold. Thus, the model both captured direct cell-cell interactions between cancer cells and stromal fibroblasts and important cell-ECM interactions. Based on the initial analysis of gene expression patterns, the described 3D breast tumor model enables the study of processes such as tumor invasion/migration, CAF formation, ECM remodeling, angiogenesis and alteration of tumor metabolism. The genetically engineered down- or upregulation of a particular gene in the cancer cells or fibroblasts of the model would provide an easy, accessible and predicable tool to further extend the knowledge of tumor biology. Moreover, the addition of immune cells or endothelial cells to the model could augment the complexity of the system, which could facilitate the generation of even more accurate representations of the tumor microenvironment.

## SUPPLEMENTARY MATERIALS FIGURES AND TABLES



## Abbreviations

2D: two-dimensional
3D: three-dimensional
CAF: cancer-associated fibroblast
*Cav1*: Caveolin 1
CLSM: confocal laser scanning microscopy
CM: Conditioned media
*Col1a1*: Collagen I
*Col4a1*: Collagen IV
*Ctnnb1*: β-catennin
Dox: doxorubicin
ECM: extracellular matrix
EMT: epithelial-mesenchymal transition
FACS: fluorescent activated cell sorting
*Fn1*: Fibronectin 1
GFP: green fluorescence protein
*Hif1a*: Hypoxia inducible factor 1
*Il6*: Interleukin 6
*Lamb1*: Laminin B1
MACS: Magneticactivated cell sorting
*Mmp9*: Matrix metaloproteinase 9
PE: polyethylene
RT-PCR: reverse transcriptase-polymerase chain reaction
*S100a4*: S100 calcium-binding protein A4
*Snai2*: Snail family transcriptional repressor 2
TAM: tumor-associated macrophage
TCP: tissuee culture plate
*Tgfb1*: Tumor growth factor β
TME: tumor microenvironment
*Tnc*: Tenascin
*Tubb*: β-tubulin
*Vegfa*: vascular-endothelial growth factor A
*Vim*: Vimentin
