# Supplementary material for: Heterotypic breast cancer model based on a silk fibroin scaffold to study the tumor microenvironment
Source: Oncotarget. 2017 Dec 22;9(4):4935–50. doi: 10.18632/oncotarget.23574 (PMC5797024; doi:10.18632/oncotarget.23574)
Supplement: Supplementary file 1 [file oncotarget-09-4935-s001.pdf]

Heterotypic breast cancer model based on a silk fibroin scaffold to study the tumor microenvironment

SUPPLEMENTARY MATERIALS

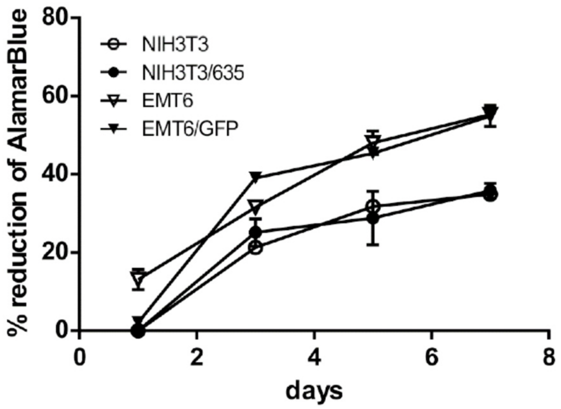

Supplementary Figure 1: Proliferation of modified cells (NIH3T3/635, EMT6/GFP) compared with the proliferation of unmodified cells (NIH3T3, EMT6) on the silk scaffolds as measured by AlamarBlue assay. The results are presented as the means of three experiments ± SEMs.

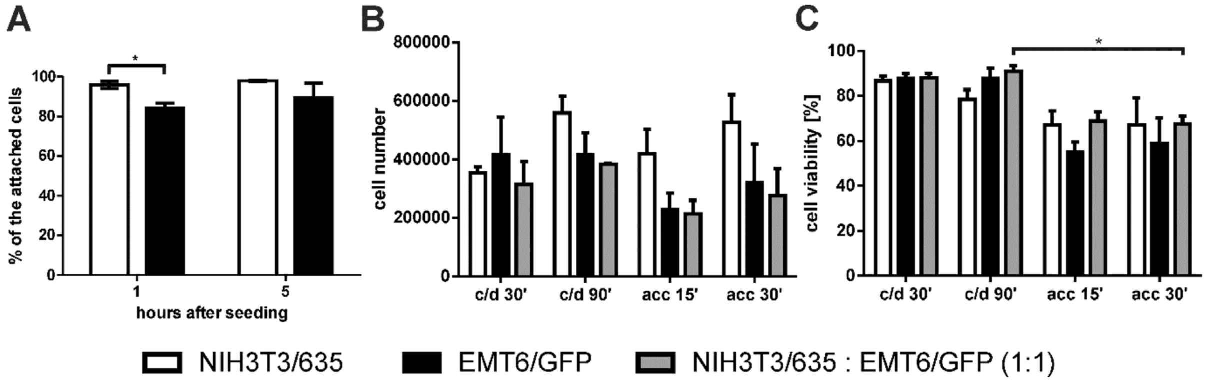

Supplementary Figure 2: Analyses of attachment and detachment of cells to scaffolds. (A) Analysis of cell attachment to the silk scaffolds as measured by counting of non-attached cells 1 and 5 h after seeding. **(B, C)** Analyses of cell detachment from silk scaffolds. NIH3T3/635 and EMT6/GFP cells were seeded on the silk scaffolds in mono-cultures or as a co-culture at a 1:1 ratio. (B) Quantity and (C) viability of cells detached using collagenase/dispase (c/d) solution or Accutase solution (acc) as analyzed after 15, 30 and 90 min using a hemocytometer counting chamber and trypan blue. Results are presented as the means of three independent experiments ± SEMs.

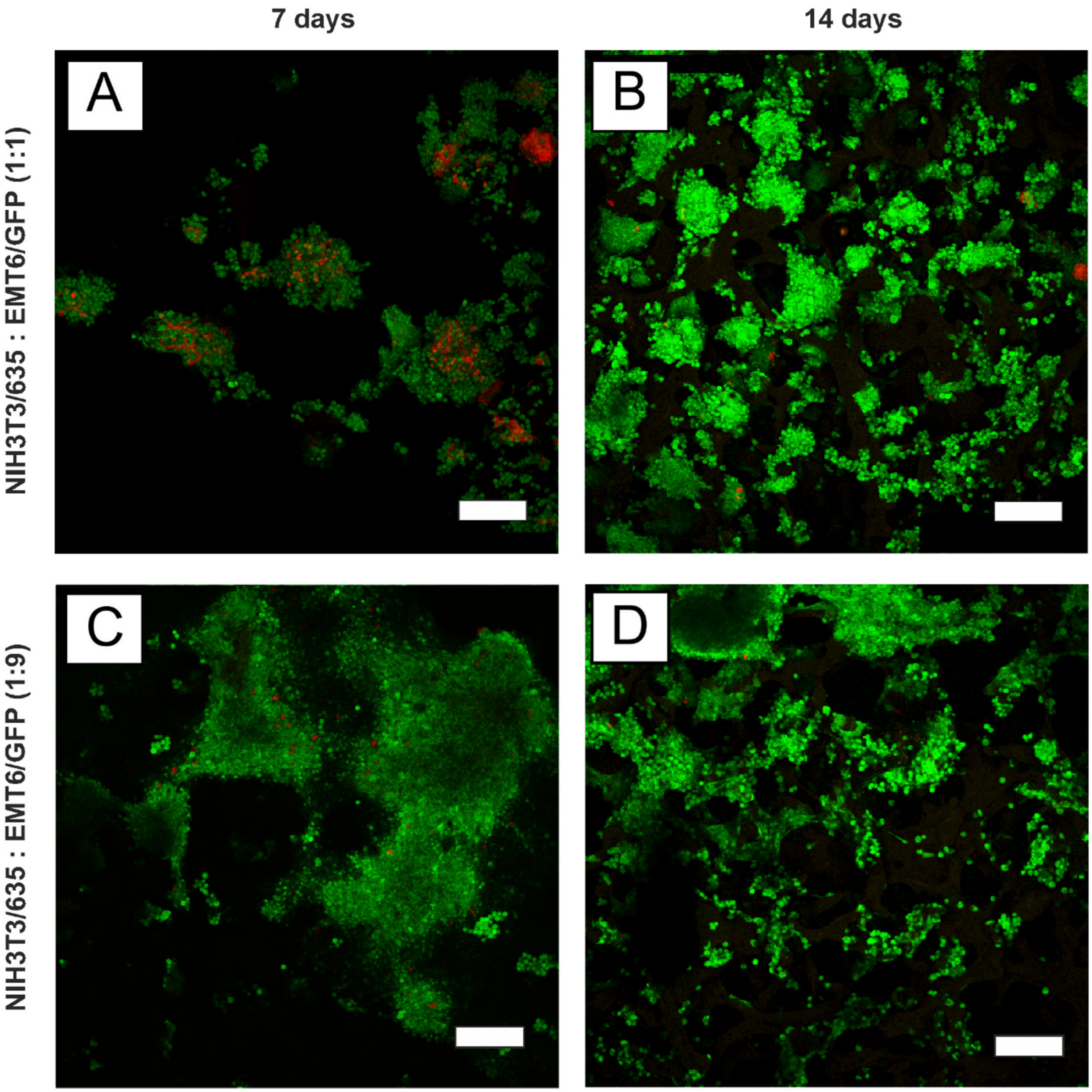

Supplementary Figure 3: Confocal images of NIH3T3/635 fibroblasts (red) and EMT6/GFP cancer cells (green) after 7 (left) and 14 (right) days of culture. NIH3T3/635 and EMT6/GFP cells were co-cultured at **(A, B)** 1:1 and **(C, D)** 1:9 ratios. Scale bar: 200 µm.

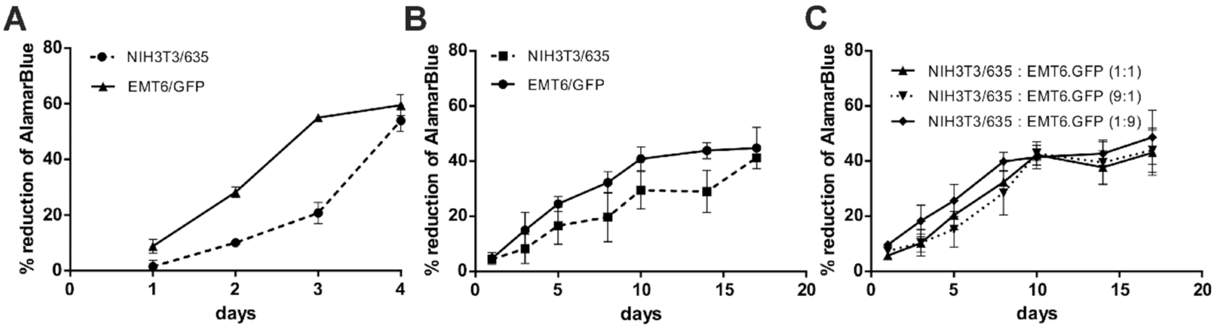

Supplementary Figure 4: Proliferation of cells cultured on tissue culture plates and on the silk scaffolds as measured by Alamar Blue assay. (A) In 2D culture, cells were tested every day for 4 days until 90% confluency was reached. **(B, C)** In 3D culture, proliferation was tested every 3 days during long term culture. Proliferation of cells in (B) mono- and (C) co-culture in various cell ratios was compared. The results are presented as the means of three experiments ± SEMs.

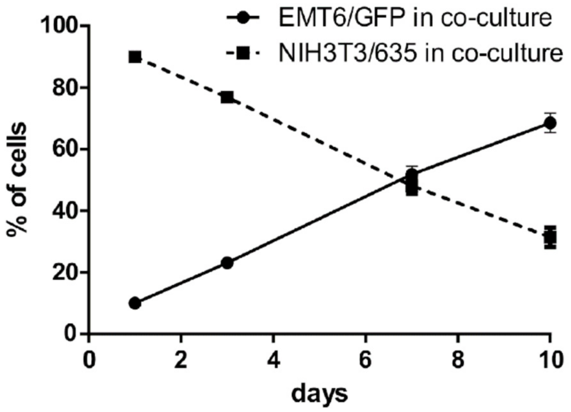

Supplementary Figure 5: Analysis of the percentages of NIH3T3/635 and EMT6/GFP cells seeded at a 9:1 ratio. Cells were detached on day 1, 3, 7 or 10 and counted using a Fuchs-Rosenthal counting chamber and a fluorescence microscope. The experiment was repeated three times; error bars represent the SEMs.

Supplementary Table 1: A list of primers and corresponding probes used for real-time polymerase chain reaction

Gene (protein)	primers	probe
Tubb **(β-tubulin)**	F: GCTGGACCGAATCTCTGTGT<br>R: GACCTGAGCGAACGGAGTC	#95
Ki67 **(KI67)**	F: GCTGTCCTCAAGACAATCATCA<br>R: GGCGTTATCCCAGGAGACT	#80
Tgfb1 **(TGF-β1)**	F: TGGAGCAACATGTGGAACTC<br>R: GTCAGCAGCCGGTTACCA	#72
Hif1a **(HIF-1α)**	F: GCACTAGACAAAGTTCACCTGAGA<br>R: CGCTATCCACATCAAAGCAA	#95
Cav1 **(caveolin 1)**	F: AACGACGACGTGGTCAAGA<br>R: CACAGTGAAGGTGGTGAAGC	#97
Acta2 **(α-SMA)**	F: GCCCTTCTTGTGCTCCATAC<br>R: GCAGCTGAACCTCCTCCTTTA	#79
Cd44 **(CD44)**	F: GTCATCAAACAGAAAGCAAGGAT<br>R: TGTTCAAGTCTTCCACCAAATG	#41
S100a4 **(S100A4/FSP-1)**	F: GGAGCTGCCTAGCTTCCTG<br>R: TCCTGGAAGTCAACTTCATTGTC	#56
Fn1 ***(*****fibronectin 1*****)***	F: CACGGAGGCCACCATTACT<br>R: CTTCAGGGCAATGACGTAGAT	#72
Tnc ***(*****tenascin C*****)***	F: GCACCCAGAGACTTTGCTTT<br>R: CAGTTGGATGTCCCCAATCT	#85
Mmp9 ***(*****MMP9*****)***	F: ACGACATAGACGGCATCCA<br>R: GTCGGCTGTGGTTCTGTTG	#83
Vegfa ***(*****VEGFa*****)***	F: GCAGCTTGAGTTAAACGAACG<br>R: GGTTCCCGAAACCCTGAG	#4
Col1a1 **(collagen I)**	F: GGCGGTTATGACTTCAGCTT<br>R: GGCCACCATCTTGAGACTTC	#83
Col4a1 **(collagen IV)**	F: TTAAAGGACTCCAGGGACCAC<br>R: CCCACTGAGCCTGTCACAC	#56
Il6 **(interleukin 6)**	F: GCTACCAAACTGGATATAATCAGGA<br>R: CCAGGTAGCTATGGTACTCCAGAA	#6
Lamb1 **(laminin B1)**	F: AAGGAGCAGCAGGAGGAAC<br>R: GGAGGTGTTCCACAGGTCAT	#79
Snai2 **(SNAI 2)**	F: CATTGCCTTGTGTCTGCAAG<br>R: AGAAAGGCTTTTCCCCAGTG	#71
Ctnnb1 **(β-catennin)**	F: TGTGGACACCCTGACTCCTA<br>R: CCGTATCCACCAGAGTGAAAA	#33
Vim **(vimentin)**	F: TGCGCCAGCAGTATGAAA<br>R: GCCTCAGAGAGGTCAGCAAA	#79
